# Specific Cationic Antimicrobial Peptides Enhance the Recovery of Low-Load Quiescent *Mycobacterium tuberculosis* in Routine Diagnostics

**DOI:** 10.3390/ijms242417555

**Published:** 2023-12-16

**Authors:** Tim J. Bull, Tulika Munshi, Paula M. Lopez-Perez, Andy C. Tran, Catherine Cosgrove, Angela Bartolf, Melissa Menichini, Laura Rindi, Lena Parigger, Nermina Malanovic, Karl Lohner, Carl J. H. Wang, Anam Fatima, Lisandra L. Martin, Semih Esin, Giovanna Batoni, Kai Hilpert

**Affiliations:** 1 Institute of Infection and Immunity, St. George’s, University of London, Cranmer Terrace, London SW17 0RE, UKkhilpert@sgul.ac.uk (K.H.); 2 TiKa Diagnostics Ltd., Cranmer Terrace, London SW17 0RE, UK; 3 St. George’s Hospital NHS Trust, Cranmer Terrace, London SW17 0RE, UK; ccosgrov@sgul.ac.uk (C.C.);; 4 Department of Translational Research and New Technologies in Medicine and Surgery, University of Pisa, 56126 Pisa, Italylaura.rindi@unipi.it (L.R.); semih.esin@med.unipi.it (S.E.); giovanna.batoni@unipi.it (G.B.); 5 Institute of Molecular Biosciences, Biophysics Division, University of Graz, Humboldstrasse 50/III, 800 Graz, Austria; lena.parigger@edu.uni-graz.a (L.P.); karl.lohner@uni-graz.at (K.L.); 6 School of Chemistry, Monash University, Clayton, VIC 3800, Australiaaxf69@psu.edu (A.F.); lisa.martin@monash.edu (L.L.M.)

**Keywords:** *Mycobacterium tuberculosis*, diagnostics, growth enhancement, cationic peptides

## Abstract

The culture confirmation of *Mycobacterium tuberculosis* (MTB) remains the gold standard for the diagnosis of Tuberculosis (TB) with culture conversion representing proof of cure. However, over 40% of TB samples fail to isolate MTB even though many patients remain infectious due to the presence of viable non-culturable forms. Previously, we have shown that two short cationic peptides, T14D and TB08L, induce a hormetic response at low concentrations, leading to a stimulation of growth in MTB and the related animal pathogen *Mycobacterium bovis* (bTB). Here, we examine these peptides showing they can influence the mycobacterial membrane integrity and function through membrane potential reduction. We also show this disruption is associated with an abnormal reduction in transcriptomic signalling from specific mycobacterial membrane sensors that normally monitor the immediate cellular environment and maintain the non-growing phenotype. We observe that exposing MTB or bTB to these peptides at optimal concentrations rapidly represses signalling mechanisms maintaining dormancy phenotypes, which leads to the promotion of aerobic metabolism and conversion into a replicative phenotype. We further show a practical application of these peptides as reagents able to enhance conventional routine culture methods by stimulating mycobacterial growth. We evaluated the ability of a peptide-supplemented sample preparation and culture protocol to isolate the MTB against a gold standard routine method tested in parallel on 255 samples from 155 patients with suspected TB. The peptide enhancement increased the sample positivity rate by 46% and decreased the average time to sample positivity of respiratory/faecal sampling by seven days. The most significant improvements in isolation rates were from sputum smear-negative low-load samples and faeces. The peptide enhancement increased sampling test sensitivity by 19%, recovery in samples from patients with a previously culture-confirmed TB by 20%, and those empirically treated for TB by 21%. We conclude that sample decontamination and culture enhancement with D-enantiomer peptides offer good potential for the much-needed improvement of the culture confirmation of TB.

## 1. Introduction

*Mycobacterium tuberculosis* (MTB), the main cause of human tuberculosis, is part of the *M. tuberculosis* complex (MTBC) that includes species like *M. bovis* and *M. africanum*. MTB grows slowly, dividing every 16–20 h in standard culture media [1]. The CDC states that a quarter of the global population has tuberculosis, with the WHO reporting 10.6 million cases and 1.6 million related deaths in 2021 [2], with TB being the leading cause of death in HIV-infected individuals. The rise of multi- and extreme drug-resistant strains (MDR- and XDR-TB) has made TB treatment increasingly difficult. Using culture methods for *M. tuberculosis* is the gold standard as it facilitates the detection and quantification of the bacteria and allows for the testing of antibiotic sensitivities or strain identification. However, only 3–4 million samples are cultured globally each year due to the necessity for specialized infrastructure and training, associated costs, and the widespread belief that MTB’s slow growth hinders a prompt diagnosis.

Antimicrobial peptides (AMPs) are short peptides (10–60 residues), predominantly cationic, with a broad spectrum of biological activity against gram-positive and gram-negative bacteria, fungi, viruses, and parasites [3]. Over 20,000 examples have already been identified, with about 3000 being from natural sources such as human defensins and cathelicidins [4]. Where it was believed that AMPs are just pore formers, in the last two decades it has become clear that they have numerous modes of action [5], including inhibiting lipid 2, acting as a cell-wall precursor essential for the bacteria [6,7] blocking the synthesis of important outer membrane proteins [8], and binding to intracellular molecules like histones, RNA, DNA [9,10], DNA-dependent enzymes [11,12,13] and ribosomes [14]. Hence, in many cases, AMPs can cross the bacterial membrane without any lytic effect and consequently reach their internal targets. Despite having some inherent weaknesses, like low-oral bioavailability, proteolytic cleavage and toxicity in humans, some are being evaluated in clinical trials [15,16].

Gram-negative bacteria sense AMPs through the PhoP/PhoQ two-component regulator system [17], whereas gram-positive bacteria have been shown to use an unrelated three-component regulator [16]. AMP sensing usually leads to modifications of the cell wall and membrane to provide resistance, however, at sublethal concentrations, some AMPs, and antibiotics in general, can stimulate growth, mobility, and a frequency of mutation and plasmid conjugative transfer [18]. For example, GL13K peptides have shown growth stimulation and an increase in metabolic activity for *Pseudomonas aeruginosa* [19]. This so-called hormetic response has been recently reported by us for slow-growing Mycobacteria (Microorganisms paper 2023). Normally, AMPs have been shown to have an antibacterial effect on slow-growing Mycobacteria, such as MTB [20].

Here we show that some AMPs at low concentrations pervade and can concentrate into the mycobacterial membrane, causing the functional disruption of unique two-component sensors normally used by MTB to maintain global transcriptomic responses retaining a dormant non-dividing phenotype. We further show that by applying these peptides during sample preparation and then further supplementing in culture, we can repress MTB’s dormancy maintenance. This leads to the stimulation of MTB growth, increasing the recovery and isolation of low-load presence in clinical samples.

## 2. Results

### 2.1. The Exposure of Mycobacteria to Some Cationic Antimicrobial Peptides Perturbs Normal Growth Patterns

**Figure 1:**
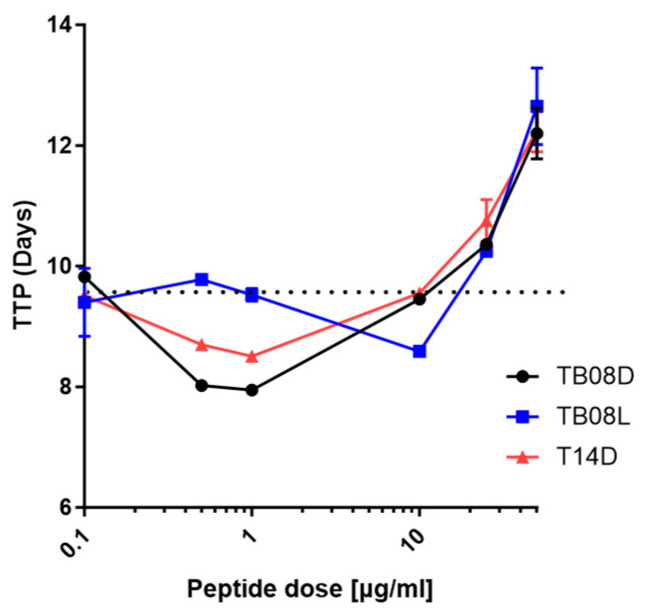
TTP (Time [Days] to Positivity [GI = 80]) vs. Dose-response curves of high load MTB (H37Rv) inoculated in MGIT medium supplemented with peptides TB08L, TB08D, and T14D, showing a 10-fold decrease in optimal dose required when using D-enantiomer. Dotted line represents average no peptide TTP for same inoculum.

In earlier studies [21,22], we identified peptides (T14D and TB08L) that exhibited a hormetic response on Mycobacteria. Many L-stereoisomeric peptides are susceptible to degradation via the proteases present within media or carried over from sample processing. The long incubation times (weeks to months) required to obtain a visible growth of mycobacterial cultures, and the need during routine culture investigations to extract from tissue samples often rich in proteases, make this a potentially limiting property. Our previous studies suggested this may be overcome by replacing the L- with D-amino acids in the peptides, affording protection due to the L-stereospecificity inherent within all natural peptidases. As predicted, when we replaced all L-amino acids with D-amino acids, maximum growth stimulation was obtained with low dosing (1 μg/mL) TB08D as compared with 10 μg/mL using TB08L. Furthermore, comparing the activity of 1 μg/mL of T14D with 1 μg/mL of TB08D, the latter showed a slightly faster Time to Positivity (TTP) of MTB at the same inoculum load, suggesting a more rapid exit from the lag phase (Figure 1). Above 10 μg/mL, all peptides became increasingly inhibitory.

### 2.2. The Peptide T14D Exposure Associated with the Disruption of the Membrane-Bound Mycobacteria Transcriptional Sensors Controlling Growing Phenotypes

We next chose to examine the differential effects of exposure to peptide T14D on the transcriptome of dormant mycobacterial phenotypes as they are stimulated to more rapidly transition through the lag phase by the peptide. The late stationary phase (35 weeks static) cultures of MTB (strain H37Rv) and an *M. bovis* cattle isolate were subcultured into standard liquid growth media with or without T14D at the concentrations previously shown to promote growth or alter membrane potential (1 and 10 μg/mL), and then aerated by shaking at 37 °C for 48 h. At this time point, there was no evidence that the subcultures had increased in number (measured by qPCR targeting mycobacterial single copy 16S rRNA) and thus, each sample was regarded as still in its lag phase. The total RNA was extracted at time points 0 and 48 h, and the cDNA was generated and applied for both species to the full MTB genome microarrays (Affymetrics Applied Biosystems, Waltham, MA, USA) which included multiple probes for each gene, spotted in triplicate. Duplicate experiments generated a data set of gene expression profiles (Supplementary File S1, Table S1) which were normalised, and multiple probes located for individual Open Reading Frames (ORF) were averaged to generate a single expression output for each gene (GeneSpring, Agilent, Santa Clara, CA, USA). Sets of transcriptomic profiles (fold change in expression relative to time 0) at 48hr with and without T14D treatment were generated from the total genome genesets of MTB (3829 genes) and bTB (3779 genes). These were then sorted into subsets of genes exhibiting a >2-fold induced or >2-fold repressed expression after 48 h of treatment with 1 or 10μg/mL of T14D either relative to the baseline (prior to dosing) at 0 h (Geneset-1i and 1r: MTB with 0 μg/mL, Geneset-2i and 2r: MTB with 1 μg/mL, Geneset-3i and 3r: MTB with 10 μg/mL, Geneset-4i and 4r: bTB with 0 μg/mL, Geneset-5i and 5r: bTB with 10 μg/mL), or a >2-fold induced or >2-fold repressed but relative to untreated control cultures after subculture in peptide-free media for 48 h (Geneset-6i and 6r: MTB 0 μg/mL vs. 1 μg/mL, Geneset-7i and 7r: MTB 0 μg/mL vs. 10 μg/mL, Geneset-8i and 8r: bTB 0 ug/mL vs. 10 μg/mL; Supplementary File S1, Table S2).

**Figure 2:**
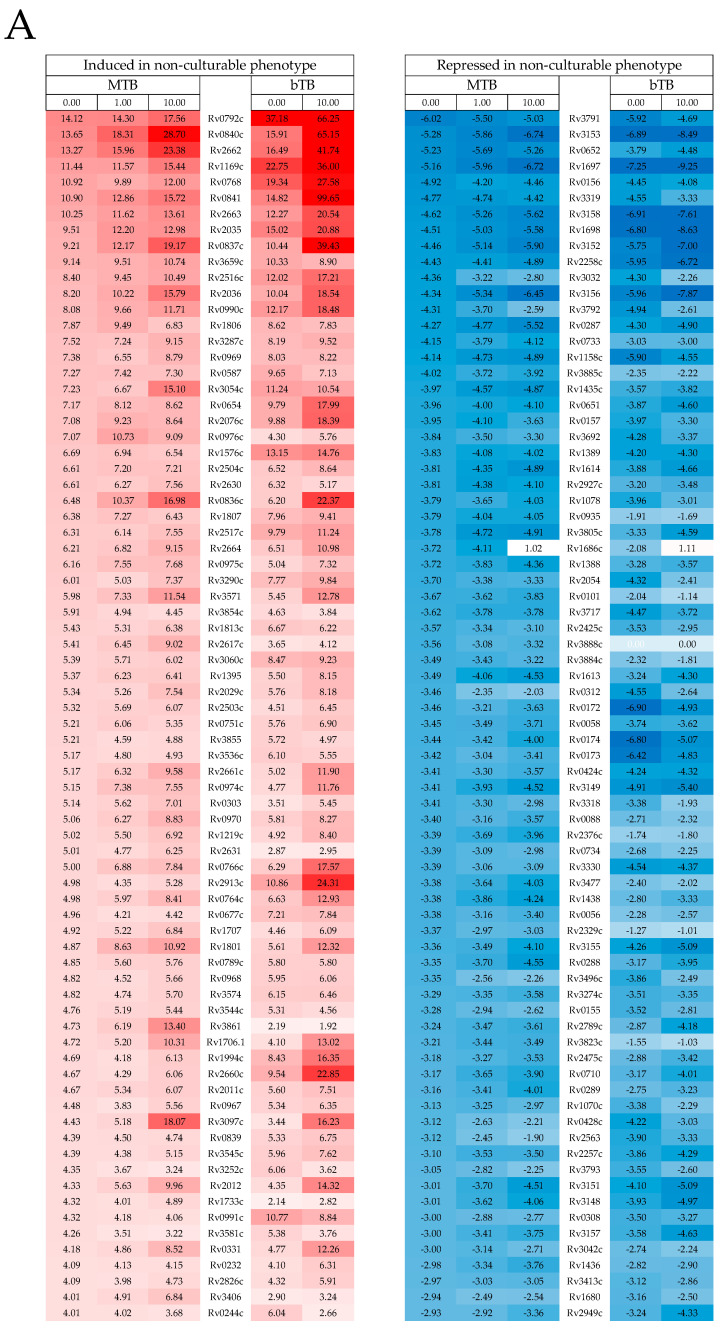

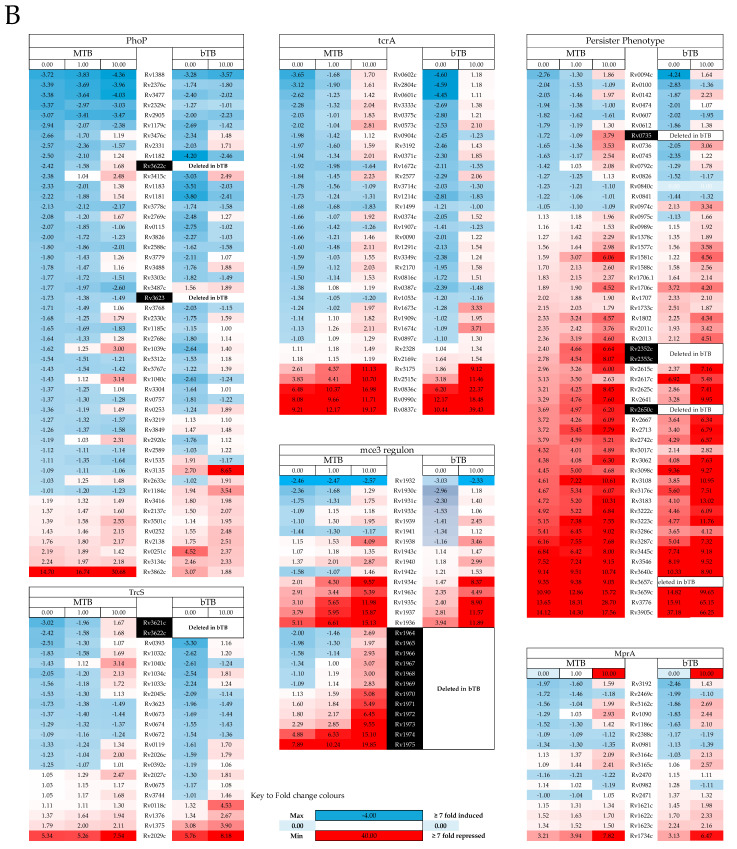
Heat maps depicting similarity in patterns of differentially expressed genes in MTB and bTB and dose-related fold changes in expression when treated with 0, 1, and 10 μg/mL of T14D (MTB) and 0, 10 μg/mL (bTB) for 48 h. Colour scale = ≥7-fold induced (fold negative values) after 48 h (blue) to >7-fold repressed (fold positive values) after 48 h (red): no change (white); (**A**) Genes known to maintain non-culturable phenotype; (**B**) two-component sensor regulons (MprA, TrcS, TcrA, PhoP), mce3 and MTB persister phenotype profiles. See text for references describing functional Genesets. Gene orthologue designations for MTB H37Rv are only displayed.

Induced expression profiles: The Geneset-1i (MTB) and Geneset-4i (bTB) representing control cultures (subcultured from stationary into new media without Peptide 14D) exhibited a >2-fold increase in expression during the 48 h in 19.7% of the total MTB genome complement, and 28.4% of the total bTB genome complement, respectively (with 69.8% overlap). This suggested that each of the test cultures was not transcriptionally dormant and had responded similarly to the subculture by 48 h. Significantly, the Geneset-3i (MTB) and Geneset-5i (bTB) representing cultures exposed to 10 μg/mL T14D for 48 h showed that significantly less (11.5% (MTB) and 14.0% (bTB)) of the total genome complement had been induced, suggesting a peptide-specific effect. We then applied hypergeometric distribution probabilities to test for the similarity of expression of each Geneset described above to a range of published MTB/bTB differential transcriptomic profiles generated under selective growth/treatment conditions (Table 1). This revealed a significant association, that increased as T14D dosing increased, in all genesets associated with upregulating and maintaining an aerobic metabolism profile [23] and a reversion of the induced/repressed gene expressions normally associated with a non-culturable MTB phenotype [24]. Comparisons of fold change expressions relative to the control and T14D dose showed 68% of genes within the aerobic metabolism profile, 39% of genes normally downregulated in the non-culturable phenotype, and 74% of genes normally upregulated in the non-culturable phenotype also showed a reversal of expression that increased with the T14D dose in both MTB and bTB (Figure 2A and Supplementary File S1; Heatmap—Geneset3i subset aerobic metabolism). These results suggest that T14D exerts a dose-dependent and more profound switch than controls towards both the aerobic metabolism and the reversion of expression profiles normally maintaining a non-culturable phenotype. A change consistent with the growth promotion characteristics and reductions in lag phase induced by T14D we observed during culture.

Strikingly, however, comparing the expressions of MTB genes after 1 μg/mL of T14D at 48 h with the control 0 μg/mL of T14D at 48 h (null Geneset-6i) returned no genes significantly (>2-fold) increased in expression as a direct response to this low dose of T14D. Even at a higher dosing of 0 vs. 10 μg/mL of T14D at 48 h in MTB, increased expression (>2-fold) was only observed in seven genes (Geneset-8i) and for 0 vs. 10 μg/mL of T14D at 48 h in bTB, only two genes (Geneset-7i), none of which were orthologues. This suggested that T14D’s mechanism of reactivity at this early time point does not involve the rapid stimulation of specific gene induction profiles, a characteristic often observed in environmental and drug treatment stress or shock responses through the activation of cell-wall receptors [25]. Of the nine genes that were significantly upregulated by the T14D, only one operon (Rv3160c-Rv3161c), associated with the initiation of aerobic growth in MTB [26], could be identified.

Repressed expression profiles: In contrast, the proportion of total genes repressed at >2-fold between 0 and 48 h with T14D treatment compared to control cultures increased markedly (10.0% to 18.4% in MTB: Genesets-1r and 3r and 11.3% to 17.4% in bTB: Genesets 4r and 5r), indicative of a broad repressive transcriptomic response to T14D by both the MTB and bTB. The relative expressions of 0 vs. 10 μg/mL of T14D treated cultures showed 698 MTB genes (Geneset-7r) and 767 bTB genes (Geneset-8r), including 583 (83% of Geneset-7r) orthologues between species, and had significantly (>2 fold) greater repression at 48 h. Furthermore, in the MTB, the degree of each of these reductions increased with the T14D dose, suggesting this response was T14D-specific and dose-dependent (see Supplementary File S1; Heatmaps; Geneset-7r). Within these 586 repressed orthologue subsets was a smaller 125 orthologue subset (Geneset-9) which was conversely induced (>2-fold) in control cultures containing no T14D (Supplementary File S1, Table S2). The hypergeometric distribution probabilities (Table 1) showed a significant association of these Genesets with the transcriptomic profiles involving networks controlling the reduction of the MTB enduring hypoxic [27] and starvation responses [28], the maintenance of the MTB antibiotic persister phenotype [29] and lipid metabolism (mce3 operon) [30], plus a repression of the sigI and whiB5 regulons associated with the re-activation of MTB from chronic infection. These complex diversities in large numbers of transcriptional profiles associated with T14D treatment suggested that a range of pathways/mechanisms, normally upregulated during early stages post-subculture were being repressed. We hypothesised that the induction of these highly varied effects by T14D could be achieved by imparting dysfunctionality to a number of sensory mechanisms. We thus further interrogated Genesets for significantly associated links between T14D reactivity, bacterial sensor genes, and their gene pathway networks (regulons). We recognized 85 genes (Geneset-10, Supplementary File S1, Table S2) that were significantly repressed by T14D related to regulons directly controlled by two-component transcriptional regulators including dosS and the dormancy-related dosR regulon [31], MtrA and cholesterol-usage pathways [32], and two important sensors, TcrA [33] and PhoP [34], controlling the initiation and maintenance of bacterial proliferation (Figure 2B and Supplementary File S1, Heatmaps).

The TcrA regulon is controlled by a three-component membrane-bound Type IV kinase signalling sensor (Rv0600c–Rv0603c) unique to mycobacteria that requires assembly at the cellular membrane for activation. It is linked to the control of the mce3 operon, which is also significantly affected in these experiments, plus the cell-wall component synthesis, cholesterol metabolism, and Fe^3+^ usage. The PhoP regulon (Rv0757; PhoP—Rv0758; PhoR) has been strongly linked to growth, cord formation in MTB, and virulence. PhoP homologues in enterobacteria have also been shown to be targets for activity by cationic antimicrobial peptides or defensins at low concentrations [35]. The disruption of PhoP control could relate to our observation (Supplementary File S2 Figure S1) that BCG cultures grown in T14D-supplemented liquid media failed to cord. In summary, these data are indicative that the T14D exposure, possibly through the inactivation/disruption of mycobacterial membrane-bound sensors, was specifically and dose-dependently influencing the transcription of both MTB and bTB in a manner concomitant with effecting a more rapid exit from profiles associated with maintaining persistent and/or non-culturable phenotypes in long-term stationary (hypoxic) cultures after subculture into aerated media. This effect is consistent with the observed reduction in the lag phase seen in the T14D-treated subcultures.

### 2.3. Cationic Antimicrobial Peptides Stimulating Mycobacterial Growth Permeate and Disrupt Artificial Prokaryotic but Not Eukaryotic Cell Membrane Models

Our transcriptomic investigations suggested a differential peptide activity linked to the disruption of mycobacterial sensors reliant on their normal functionality on intact cell membranes. We, therefore, investigated the binding and disruption of function (leakage) of selected peptides when applied to artificial eukaryotic or prokaryotic cell membranes and their direct effects on the maintenance of membrane potential by mycobacterial membranes in culture.

Using Quartz Crystal Microbalance with Dissipation Monitoring (QCM-D), we measured the interactions between peptides and different model membranes. We compared selected growth-stimulating peptides (T14L, T14D, and TB08L) and a control peptide (D02D) consisting of D-amino acids shown to inhibit MTB growth even at the lowest concentration tested (0.24 µM). The peptides were applied at various concentrations onto an artificial membrane layer, created via the rupture of liposomes onto a quartz sensor [36,37,38] designed to represent a eukaryotic (POPC) bilayer and a more complex (POPC:POPG [4:1]) lipid mixture similar to a prokaryotic cell membrane. These experiments show the binding of the peptide to the membrane as a temporal change in frequency (Δf) and the associated change in viscoelasticity of the resulting peptide–membrane layer (ΔD).

**Figure 3:**
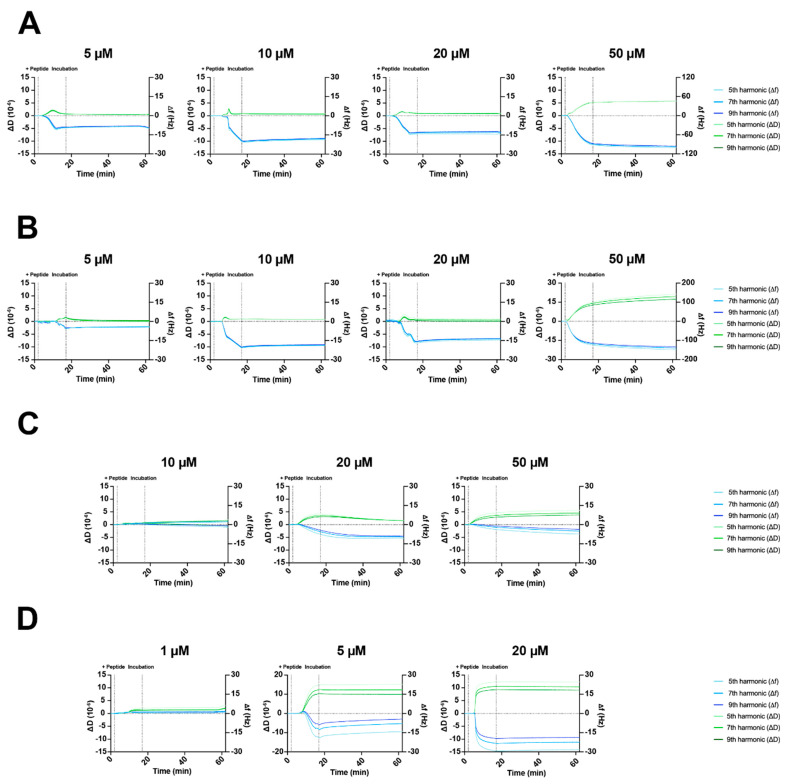
Representative QCM-D membrane interaction (Δf (Hz)) and membrane viscoelasticity (ΔD (10^−6^)) time profiles at the 5th, 7th, and 9th harmonics for 5–50 μM of peptides (**A**) T14L, (**B**) T14D, (**C**) TB08L, and (**D**) Control peptide D02D applied to 4:1 POPC:POPG bilayers for 15 min (dotted line) followed by washing for 45 min (a full set of profiles with other membrane models are available in Supplementary File S2).

On the eukaryotic model membrane, the T14L, T14D, and D02D showed little or no binding to the membrane. However, interestingly, the TB08L did bind the zwitterionic POPC membrane at low peptide concentrations (1–5 μM) which resulted in the disruption of the lipid bilayer at the higher concentration of 20 μM (Supplementary Figure S3). In contrast, very different peptide–membrane interactions were evident if a prokaryotic model membrane was used (Figure 3).

For the peptides T14L and T14D, based on the shape of the Δf vs. time and ΔD vs. time graphs, the peptide interaction with the membrane appears to vary at concentrations of 5–10 μM. At the higher concentration of 20 μM, no further peptide binding is evident. Effectively, there is little or no change in the dissipation during the peptide binding and the overlapping harmonics indicating that there is an even distribution of the peptide throughout the membrane layer [37,39,40]. However, at the highest peptide concentration (50 μM), a large amount of peptide interacts with the membrane, with a Δf of −100 Hz for the D14L and −140 Hz for the D14D peptide bindings and the concomitant ΔD values also increased significantly (increased viscoelasticity), although the harmonics remain overlapping, suggesting that the peptide is evenly distributed across the bilayers (Figure 3A,B).

For peptide TB08L, there were some similarities to the actions of the T14L and T14D on the POPC:POPG membranes, with a concentration-dependent response in Δf detected between 10 and 20 μM and approximately overlapping harmonics seen; however, no significant change in either the Δf or in the ΔD was observed at 50 μM (Figure 3C). In general, the mechanistic characteristics are consistent with those of the T14L and T14D peptides, supporting that the peptide insertion is evenly spread across the lipid bilayers.

The response for the control peptide D02D is quite different on the POPC:POPG membrane (Figure 3D). There are little or no peptide binds at 1 μM, however, a concentration-dependent response is observed at higher concentrations (5 and 20 μM), with Δf values of −15 and 30 Hz, respectively, for the 5th harmonic. The differential response across the three harmonics for both the Δf and ΔD parameters is consistent with the peptide binding the membrane bilayers in a surface [37,39,40]. Interestingly, even with the highest concentration of peptide added to the membrane, no disruption to the bilayer was observed.

In summary, all peptides were able to interact with the POPC:POPG membrane and in a concentration-dependent manner. The observed trend from T14L, T14D, and TB08L data was that these peptides associated with the membrane, in a transmembrane-like manner at concentrations up to 20 μM and continued to bind in this manner at 50 μM for T14L and T14D, albeit with an impact on the bilayer, creating a more viscoelastic surface. The non-stimulating control D02D peptide was unable to incorporate into the POPC:POPG layer but created a carpet-like peptide-rich surface and may contribute to a physical barrier leading to the growth inhibition observed in the earlier data.

### 2.4. Cationic Antimicrobial Peptides Stimulating Mycobacterial Growth Cause the Leakage of Prokaryotic Bilayers While an Inhibitory Peptide Does Not

**Figure 4:**
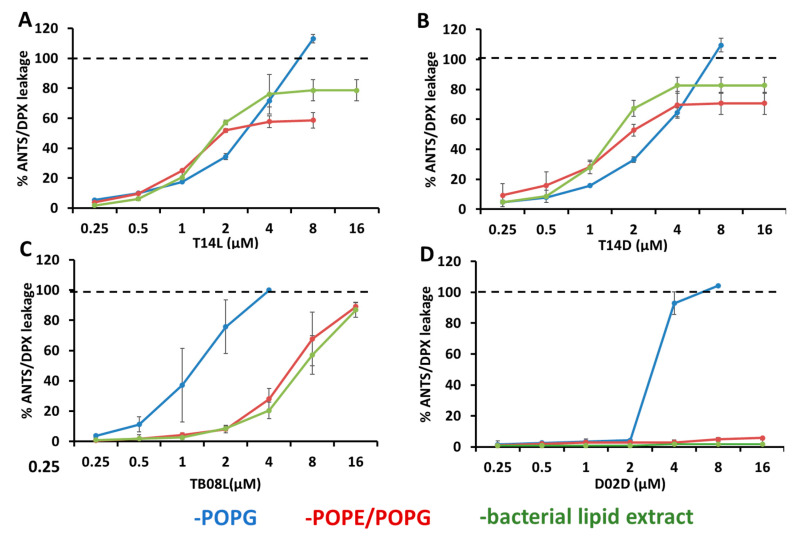
Leakage of liposomes by peptides. The fluorescence of ANTS from liposomes containing POPG (blue), POPE/POPG (3:1) (red), and bacterial lipid extracts (green) was obtained while adding increasing amounts of (**A**) T14L, (**B**) T14D, (**C**) TB08L, and (**D**) D02D peptide, leading to peptide concentrations in the cuvette from 0.25 to 16 μM, corresponding to a lipid-to-peptide molar ratio from 200:1 to 3:1. An amount of 100% leakage (dashed line) was derived by adding 10% Triton X-100. Experiments were conducted twice, and the reported results represent the means of these experiments, along with their corresponding standard deviations.

Leakage experiments were performed on liposomes containing POPG (high anionic procaryote model membrane), POPE/POPG (3:1) (low anionic prokaryote model membrane), and bacterial lipid extract (natural prokaryote model membrane, low anionic). The growth-stimulating peptides T14L, T14D, and TB08L, as well as the non-stimulating control peptide D02D, were used in a concentration-dependent manner. As expected, all peptides caused leakage in the high anionic procaryotic model membrane and with peptide D02D only at higher concentrations (Figure 4). Interestingly, only the growth-stimulating peptides induced leakage at the low prokaryotic model membranes, whereas the inhibitory peptides only showed negligible leakage at the highest concentration for both low anionic prokaryotic model membranes, in combination with POPE/POPG (3:1) or bacterial lipid extracts, respectively.

**Figure 5:**
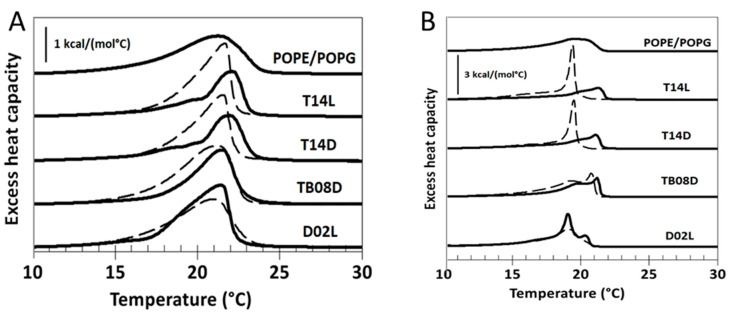
Differential Scanning Calorimetry (DSC) of POPE/POPG 3/1 mol/mol. Scan rate: 0.5 °C per minute. The thermogram at the top relates to the lipid mixture only; full lines: lipid:peptide 25/1 mol/mol, dashed lines: lipid:peptide 100/1 mol/mol. (**A**) Heating scans, (**B**) cooling scans.

In addition to the leakage experiments, differential scanning calorimetry (DSC) was performed using the same peptide set (T14L, T14D, TB08L, D02D). Where, in the heating scans (Figure 5A), all peptides show a similar change in the phase behaviour, the cooling scans reveal a very different behaviour between the growth-stimulating and the control peptide. At a low peptide/lipid ratio, peptide D02D shows a very similar phase transition to POPE/POPG alone. At a higher ratio, changes in phase behaviour occur compared to the POPE/POPG alone, which are distinctively different to the growth-stimulating peptides. These results confirm the differences between peptides seen in the leakage experiments using POPE/POPG. The phase transition of pure POPE/POPG is noticeable at 21.2 °C during heating scans and at 19.7 °C during cooling scans (see Figure 5B). The peptides that stimulate growth cause a shift in the phase transition temperature towards higher values. This behaviour can occur when the peptides preferentially interact with negatively charged phospholipids, leading to lipid separation within the POPE/POPG mixture. As a result, the transition temperature shifts closer to that of pure POPE, which is at 25 °C because the POPE was not hindered from the transition. Of note was that the transition temperature of pure POPG is −5 °C, see [41] for details. One plausible explanation for this is that these peptides temporarily reorganize the membrane upon insertion, causing it to become leaky. Additional evidence supporting this hypothesis comes from the distinct behaviour of fluorescence curves in the leakage assay (Supplementary Figures S4 and S5). While growth-inhibiting peptides induced a gradual increase in the fluorescence of incorporated dye ANTS over time, this increase in ANTS fluorescence was only observed for a short duration with all growth-inhibiting peptides, suggesting an open and close mechanism.

It is important to note that the DSC and leakage assay results may not always be directly correlated. A similar effect has been observed with the antimicrobial peptide OP-145 which was found to disorder the lipid packing of POPE/POPG without inducing leakage in this membrane composition [41]. Indeed, its membrane activity was confirmed by the significant depolarization of the bacterial membranes in *E. coli*. This effect, rather than an increased membrane permeability, likely contributed to its antimicrobial activity.

### 2.5. Cationic Antimicrobial Peptides Stimulating Mycobacterial Growth Reduce the Mycobacterial Membrane Potential, Which Can Then Be Maintained by Continued Low-Peptide Dosing

**Figure 6:**
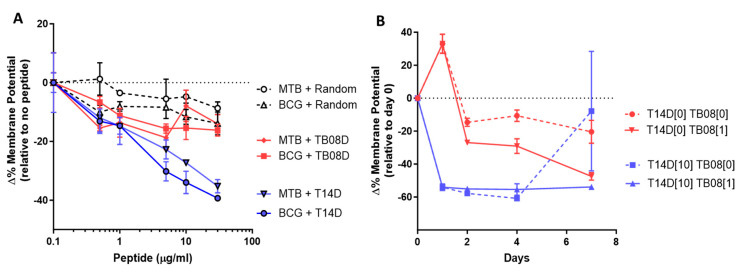
Membrane potential assays. (**A**) Changes in MP of MTB cultures after dosing with peptide T14D, peptide TB08D, or RAND peptide mix (0.5−50 μg/mL) after 3 h. Levels are displayed as a differential relative to a no-peptide control. (**B**) Changes in membrane potential of mycobacterial culture after subculture into TiKa-Kic medium with or without 10 μg/mL of T14D, followed by growth in 7GCO+ growth medium supplemented with or without 1 μg/mL of peptide TB08D.

To investigate the effect of selected peptides directly on the mycobacterial membranes, we monitored changes in membrane potential (MP) through the ability of intact polarised membranes to maintain the uptake of a cell wall-penetrating dye (DiOC_2_) (Figure 6A). An 11-residue randomised sequence D-peptide mix (RAN-D) exerted a minimal reduction in MP relative to the no peptide controls, whilst a 15% decrease was observed with a 1 μM of T14D, and up to 40% with a 50 μM of T14D after 3 h. The TB08D concentrations >1 μM, however, only induced a mild depolarization that maximised at a 15% reduction in MP, relative to no peptide. We then tested the sequential exposure to T14D followed by TB08D (Figure 6B). The MTB or *M. bovis* BCG cultures (grown to stationary phase) were initially exposed to a standard bacterial culture broth (TiKa-Kic medium), with or without T14D for 18 h, washed by pelleting, then transferred into 7GCO+ culture medium (with or without TB08D) for 6 days. The TiKa-Kic medium without T14D induced a transient positive change in the MP that gradually diminished on subculture alone. Replacing this with TB08D at the culture stage increased the speed of this restoration consistent with a gradual incorporation of the peptide. Contrastingly, the initial exposure to TiKa-Kic + T14D induced a rapid decrease in the MP which was maintained after washing and subculture into 7GCO+ for about four days before MP was restored to normal levels. The sequential treatment with TiKa-Kic + T14D, washing, and then incubation in 7GCO + TB08D, maintained the low MP throughout the seven days.

In summary, all the presented data show that (1) only a few antimicrobial peptides are capable of growth stimulation in slow-growing Mycobacteria, (2) the growth-stimulating peptides tested are probably inactivating or disrupting the mycobacterial membrane-bound sensors, and consequently activating the gene pathways to rapidly exit the lag phase, (3) such peptides can integrate into prokaryotic model membranes and cause leakage, (4) on MTB cultures, those peptides caused a change in the membrane potential that is reversible, and (5) D-enantiomer variants require less concentration for the same growth stimulation effects, however, L-enantiomers are still effective at higher concentrations.

### 2.6. The Incorporation of Cationic Antimicrobial Peptides into Routine Sample Processing Protocols Enables a Greater Recovery of MTB from Tuberculosis Samples

We hypothesised that the properties of our discovered peptides were conducive to improving the conventional methodology for recovering and isolating MTB from clinical samples. We thus designed a novel method (Index) of sample decontamination (based on the high antimicrobial properties of T14D against non-Mycobacteria) followed by culture in conventional liquid growth media supplemented with TB08D (based on its efficacy in reducing the lag phase and recovering from the stationary phase). We then evaluated this novel technique using a parallel split sample trial compared against a conventional standard NaOH decontamination-based method (Reference). All culture isolates were identified by either in-house tests or reference laboratory confirmatory identification. Recruitment was carried out at two separate sites and successful sample provision was obtained from 155 patients ranging from 1 to 86 years. We obtained 255 samples, of which 253 were successfully processed (blinded) and cultured in parallel including 119 sputa, 20 bronchial lavages, 44 faeces, 53 urines, 10 fine needle aspirates, 3 pleural fluids, 1 biopsy, 1 synovial fluid, 1 ascitic fluid, and 1 Cerebral Spinal Fluid (CSF). A retrospective diagnosis was available from 111/155 (72%) patients, of which 37/111 (33%) had a diagnosis unrelated to tuberculosis (seven of which grew non-tuberculous mycobacteria by the Index method). Three patients (3%) were MTB contacts and 71/111 (64%) had possible or confirmed Tuberculosis including 29 patients (26%) with active pulmonary TB, 3 patients (2%) with resolved TB disease at the time of sampling, 25 (22%) patients with non-pulmonary TB, 12 (11%) with disseminated TB, and 5 (4%) with latent TB reactivity.

An amount of 30/56 MTB isolates were grown from the same sample by both the Index and Reference methods [Supplementary File S1: Table S4]. Significantly, however, the Index method increased the sample positivity rate by 46% (26 MTB isolates, including one MDR-TB from faeces). This included a subset of 9/56 (16%) samples that grew in the Index method but failed to decontaminate by the Reference Standard method. Overall, the Index method isolated at least one isolate of MTB when the reference standard failed from 6/29 (20%) patients with a previously culture-confirmed pulmonary TB (overall an improvement of 5%), 4/19 (21%) from patients with a TB diagnosis treated empirically (overall an improvement of 16%), and, surprisingly, one isolate from the faeces of a patient with latent TB. In this trial, we were expecting the gold standard to be imperfect. To estimate the validity of false negatives, we, therefore, applied a resolver methodological approach [42] which expected MTB-positive isolation in a designated subset of the total samples from patients with an available clinical history, defined as having a medium or low expectation of receiving an expected culture confirmation result based upon clinical diagnosis at the time of sampling, an administered therapy, a retrospective outcome, or a previous culture history in a related sample (listed in Supplementary File S1 Table S4). This showed 25/42 (60%, Cl 43.2–74.4) positive samples in the reference method data set and 33/42 (79%, Cl 63.2–89.7) suggested an increase in test sensitivity of 19% by the Index method (Supplementary Figure S2). The sample Time to Positivity (TTP, in days) was taken from the growth index standard cut-off as designated by the BACTEC MGIT320 automated culture machine version 6.01B (Becton Dickinson, Oxford, UK). The MTB was isolated faster via the Index method in 21/30 (70%) samples. Two fine needle aspirate (FNA) samples were significantly faster via the Reference method. The MTB TTP from sputa using the Index method averaged 10 days (Range 2–23 days) compared with 14 days (Range 3–70) by the Reference method. No samples were solely MTB-positive via the Reference method. Significantly, 10 sputum samples were MTB-positive only via the Index method, with these averaging a TTP of 23 days (Range 7–60), which is lower than the average for other TTPs, suggesting low bacterial loads may have been present in these samples (Table 2).

A Cox two-sided log-rank test on the complete dataset with the Index Test being less likely to ‘survive positivity’ than the Reference Standard was statistically rejected with high significance (*p* < 0.0001), with median ‘survival positivity’ at 16 days for the Index and 23 days for the Reference Standard (Ratio 0.6809: 95% CI 0.44 to 1.05) (Supplementary Figure S1), indicating that the Index method was significantly more rapid than the Reference method in this study.

## 3. Discussion

In this study, we describe, characterise, and show the utility of two short cationic peptides, T14D and TB08D. In previous studies [21,22], we have shown a growth-stimulating effect of T14D on MTB, *M. bovis*, *M. marinum*, *M. avium*, *M.intracellulare*, *M. celatum*, and *M. abscessus* within a narrow activity range of 0.1–1 µg/mL, an ability to stimulate a mean 29-fold increase in recoverability, and improved sensitivity of up to three logs when compared with conventional cultures of *M. avium* subsp. *Paratuberculosis* (MAP) from cattle. Antimicrobial activity had been previously observed in T14L and T14D against gram-negative and positive bacteria [43,44], but when tested against mycobacteria we found only an inhibition of growth occurred at higher concentrations. Intriguingly, however, we observe here that at relatively low concentrations, each of the four peptides (T14D, T14L, TB08L, and TB08D) stimulated mycobacterial growth, reducing the lag phase on subculture from long-term stationary phase cultures. We showed that this phenomenon was dose-dependent and occurred at lower concentrations for D-stereo enantiomers relative to L-stereo enantiomers, with all peptides increasing in toxicity at higher concentrations. We further demonstrated that these peptides engaged with mycobacterial membranes reversibly, but when supplemented into conventional mycobacterial growth media, they became gradually, but not permanently, incorporated, disrupting the membrane potential at low concentrations, stimulating growth and, at higher doses, inhibiting growth possibly as a result of membrane damage leading to leakage, as observed in our model membrane system. In contrast, a control peptide with no growth stimulatory activity showed no leakage in a low-anionic prokaryotic model membrane and a diverse phase behaviour when measured by DSC, suggesting a different mode of interaction.

Using full MTB genome microarrays, we demonstrated that exposure to T14D induced profound but similar effects on the transcriptome of both MTB and the related animal pathogen bTB. Using starter cultures predominantly including dormant/persister phenotypes subcultured into conventional aerated media, we show that T14D is specifically, and dose-dependently, associated with a decreased expression of the same genesets in MTB and bTB used to maintain a non-culturable dormant and persistent phenotype, and an up-regulation of genesets controlling aerobic metabolism and expression profiles aligning with a viable growing phenotype. Significantly, those differentially expressed genesets most associated with T14D exposure could be assigned as under the control of two-component sensor mechanisms located in the mycobacterial membrane (TcrA, MprA, and PhoP) which each rely upon an intact membrane structure for the functionality/activation of their signalling components. We hypothesize that T14D incorporation into the mycobacterial membrane at low concentrations builds and either through the disruption of structure or membrane potential, affects membrane-bound sensor functionality causing a reduction in response signalling that leads to the repression of their associated regulons. Our observations of the reversible nature of peptide penetration into mycobacterial membranes suggest that although the effect is rapid and broad-ranging, the long-term maintenance of peptide association is necessary to commit the depth/speed of conversion from the non-culturable phenotype.

The culture confirmation of MTB remains the gold standard for the diagnosis of Tuberculosis (TB) with culture conversion representing proof of cure [45]. The paradigm of MTB culture, however, is that conventional microbiological investigation methods are inefficient and so slow that they can only ever be used retrospectively [46]. Despite some improvements in the speed of colony visualisation [47], isolating and detecting colonies of MTB from samples typically takes 4–5 weeks and, in the case of dormant or low-load persistent phenotypes, not at all. As most TB samples are obtained from non-sterile sites, the selective removal of contaminating flora prior to culture is mandatory. Unfortunately, conventional sample preparation systems need to use high acid or alkali steps to decontaminate background flora, and this can also kill [48] or drive large proportions of MTB loads into anaerobic-based metabolism and dormant non-culturable states, decimating the likelihood of recovery in low-load samples [49]. Recent UK figures [50] show that despite acceptable rates of sampling, 40% of all diagnosed TB cases and 64% of non-pulmonary TB were not confirmed via culture. Most culture failures can be related to a low bacterial load within the sample. Smear-negative samples are at least twice as likely than smear-positives to generate a negative culture even when they remain infectious [51]. The persisting low sensitivity of this conventional and gold standard diagnostic has thus mostly been perpetuated by the inability of media, devised and not improved ostensibly since the 1970′s [52], to resuscitate MTB from its dormant or persistent states. We hypothesised that T14D and TB08D peptides could be used to supplement and improve conventional sample preparation and culture media that could help address this issue. We thus devised a novel two-stage methodology. Firstly, choosing to incorporate T14D, with both broad-spectrum antimicrobial activity and mycobacterial growth priming activity, into an initial background flora decontamination step, followed by supplementing TB08D, with high mycobacterial growth stimulating activity, into conventional culture media. An evaluation of this system against a conventional sample preparation and culture system (NaOH decontamination: MGIT culture) in a 255-sample study from 155 suspected TB patients showed an unparalleled increase in both the recovery rate and the speed of recovery of MTB from a range of sample types. The peptide supplementation increased the sample MTB positivity rate by 46%, test sensitivity by 19%, and decreased the MTB Time To Positivity in 70% of positives (average 7 days faster, Range 1–46 days). MTB recovery in samples from patients with a previously culture-confirmed TB increased by 20% and those empirically treated for TB by 21%. Patients with low-load infection, as indicated by smear-negative sputum samples or disseminated infections, were significantly more likely to recover and isolate MTB from samples using the peptide-enhanced method. Furthermore, the method decreased the sample contamination rate by 12%, significantly increasing the ability to recover MTB from commonly difficult-to-decontaminate faecal samples.

We conclude that the short antimicrobial cationic peptides we have described show unique abilities to kill a wide range of non-mycobacterial flora whilst interfering with mycobacterial sensing networks maintaining non-culturable phenotypes, in a manner that promotes transition to viable growing states. The application of these peptides into routine microbiological investigations significantly increases the probability of culturing from low-load conditions and offers a timely adjunct to the problem of unreliable MTB recovery from samples of patients with suspected Tuberculosis.

## 4. Materials and Methods

### 4.1. Peptides

The following peptides were used in this study (final epithet denoting either the D or L enantiomer used): T14D (NH_2_-wkivfwwrr-CONH_2_); T14L (NH_2_-WKIVFWWRR-CONH_2_); TB08D (NH_2_-lfkllGkiihhvGnfv-CONH_2_); TB08L (NH_2_-LFKLLGKIIHHVGNFV-CONH_2_); D02D (NH_2_-wkiGfdwrr-CONH_2_); and RAN-D (a randomly created 11-mer sequence of D-amino acids). Peptides were synthesized as previously reported [20]. Briefly, synthesis was performed via automated solid-phase peptide synthesis (SPPS) on a MultiPep RSI peptide synthesizer (Intavis, Tübingen, Germany) using the 9-fluorenyl-methoxycarbonyl-tert-butyl (Fmoc/tBu) strategy on TentaGel^®^ HL RAM resin. The crude peptides were dissolved in 20% (*v*/*v*) acetonitrile (ACN, Jencons-VWR, Leicestershire, UK)/80% (*v*/*v*) water containing 1% (*v*/*v*) TFA, and analysed via analytical reversed-phase (RP) HPLC on a Shim-pack VP-ODS (120 Å, 150 mm × 4.6 mm, Shimadzu, Milton Keynes, UK) using a Shimadzu LC2010AHT system (Shimadzu, Milton Keynes, UK). The identity was verified by a liquid chromatography-electrospray ionization mass spectrometry (LC-ESI-MS) Shimadzu LC2020 system (Shimadzu, Milton Keynes, UK) equipped with a Jupiter 4 μm Proteo C18 column (90 Å, 250 mm × 4.6 mm, Phenomenex, Cheshire, UK). The crude peptides were purified to a homogeneity of >90% by preparative RP HPLC on a Shimadzu LC2020 system equipped with a Jupiter 10 μm Proteo C18 column (90 Å, 250 mm × 21.2 mm, Phenomenex, Cheshire, UK). Finally, pure products were characterised using analytical RP-HPLC and LCMS. The TiKa14D was purchased (Bachem, Bubendorf, Switzerland) with a purity of >90%.

Mycobacterial culture and growth curves: The stock MTB cultures were prepared by growing MTB H37Rv (*ATCC* 25618) or *M. bovis* (strain type RD1-RD3, RD8, RD14, RD16 positive: RD4-RD7, RD9-RD13, RD15 negative: bTB; an animal isolate kindly supplied by J. Sawyer, APHA, Weybridge, UK) to their late stationary phase (30 weeks) in a 200 mL culture of standard 7GCO+ medium (4.7 g Middlebrook 7H9, 1 g casitone, 2.5 mL glycerol, 10% OADC/L (Oxoid, Basingstoke, UK)) without shaking at 37 °C. Growth curve cultures were inoculated with 10^5^ cfu/mL stock culture (estimated by OD_600nm_) into 7 mL MGIT medium (including PANTA supplement) with or without peptides at designated concentrations as described in the text.

### 4.2. Membrane Potential Studies

The mycobacterial culture stocks prepared as described above were diluted in 7GCO+ medium to 0.8 OD_600nm_, aliquoted (500 μL) into sterile reaction tubes, and incubated overnight at 37 °C. For the dose-response experiments, stock cultures were centrifuged at 1700× *g* for 15 min and pellets were resuspended in 7GCO+ medium with peptide dilutions (0.5–50 μg/mL) of T14D, peptide TB08L, or RAND peptide added then incubated at 37 °C for 3 h. The protonophore carbonylcyanide *m*-chlorophenyl hydrazone (CCCP) was used as a positive-depolarising control (10 μM). For the time course, study cultures were centrifuged at 1700× *g* for 15 min then resuspended in a simple bacterial culture medium alone (½ strength Müller-Hinton Broth: Oxoid, UK) with or without peptide T14D (10 μg/mL) and incubated overnight with gentle rotary mixing (100 rpm). The membrane potential was determined by adding 3 mM stock 3,3′ diethyloxacarbocyanine iodine (DiOC_2_(3); Invitrogen, London, UK) to a final concentration of 15 μM, incubating at RT for 20 min in the dark, washing once in PBS, then reading for dye uptake using a 480/530 nm: green vs. 488/610 nm: red ratio on a Fluostar Optima (BMG Biotec, Aylesbury, UK) in Black 96-well Costar plates (Fischer, London, UK).

The *QCM-D* Liposomes were produced from 1-palmitoyl-2-oleoyl-sn-glycero-3-phosphatidylcholine (sodium salt, ≥ 98%) (POPC) and 1-palmitoyl-2-oleoyl-sn-glycero-3-phosphatidylglycerl (sodium salt, ≥ 98%) (POPG) (Cayman Chemical, Ann Arbor, MI, USA) in chloroform (Sigma, Livonia, MI, USA) prepared as POPC or POPC:POPG in a 4:1 ratio that was dried and rehydrated in 20 mM of phosphate, 100 mM of NaCl PBS (pH 7.4) to 0.5 mM, before being extruded in a Mini-Extruder (Avanti, Weston, FL, USA) through an Avanti PC membrane (0.1 μm) at least 15 times. The extruded liposomes were diluted to 0.1 mM using 20 mM of phosphate, 100 mM of NaCl PBS (pH 7.4) for pure POPC and 20 mM of phosphate, 250 mM of NaCl PBS (pH 7.4) for POPC:POPG, then deposited on a gold sensor coated in silicon dioxide and washed with 100 mM of NaCl PBS. The QCM-D was performed using a QSense E4 (Q-Sense, Gothenburg, Sweden). Peptides were introduced at 50 μL min^−1^ for 15 min before stopping the flow and being allowed to react with each bilayer for 45 min. The membrane was then washed with 100 mM of NaCl PBS for 60 min. The change in frequency (Δf and dissipation (ΔD) were recorded for the fifth, seventh, and ninth harmonics for each experiment. The data was recorded using QSoft401 (version no. 2.5.15.671), with data being exported using QTools 3 to OriginPro 8, and was subjected to Savitsky-Golay smoothing (polynomial order: 2, points of window: 5) and normalised such that the Δf and ΔD of the lipid bilayer without peptide were 0 Hz and 0, respectively.

### 4.3. Leakage and DSC Studies

For the preparation of the liposomes used in leakage assays and DSC experiments, we employed three different lipid components: 1-palmitoyl-2-oleoyl-sn-glycero-3-phospho-(1′-rac-glycerol) (sodium salt) (POPG), 1-palmitoyl-2-oleoyl-sn-glycero-3-phosphoethanolamine (POPE), and *E. coli* Polar Lipid Extract (composed of 67.0% PE, 23.2% PG, and 9.8% CL). These lipids were obtained from Avanti Polar Lipids, Inc. (Alabaster, AL, USA) with a purity exceeding 99%.

The preparation of the liposomes followed a previously established protocol [41,53]. In brief, for the leakage experiments, we generated three distinct membrane systems: (i) liposomes composed solely of POPG, (ii) liposomes consisting of a mixture of POPE and POPG in a molar ratio of 3:1, and (iii) liposomes derived from polar lipids extracted from *E. coli*. These lipid systems were selected because they closely mimic the typical bacterial membranes [54]. Although many studies mention the presence of phosphatidylinositol in mycobacterial membranes, it remains unclear whether these species are more abundant in the cell wall or the cytoplasmic membrane, and how the phospholipid composition changes during growth. *M. tuberculosis* has a distinct membrane composition that can be modified as an adaptive mechanism for its persistence in the host [55]. Furthermore, *M. tuberculosis* has its own regulatory system for maintaining the composition of its cell wall lipids, which can vary widely among different species. To avoid the influence of this dynamic adaptive mechanism, we chose model membranes that contain the major lipid species found in mycobacterial membranes and closely resemble the key physicochemical properties of these membranes and their phospholipids, including their varying anionic content.

Initially, the lipids in powder form were dissolved in a solvent containing chloroform and methanol in a 2:1 ratio, resulting in a final lipid concentration of 20 mg/mL of lipid. Subsequently, these samples were evaporated under nitrogen steam at 33 °C for approximately 30 min and then dried overnight under vacuum conditions. The dried lipid films were stored at +4 °C until further processing. To create the liposomes, the dry lipid films were incubated with 1 mL of a fluorophore-buffer solution (containing 10 mM of Hepes, 68 mM of NaCl, 12.5 mM of ANTS, 45 mM of DPX, and a pH of 7.4) and the liposomes were formed at 65 °C with vigorous vortexing. Large unilamellar vesicles (LUVs) were then generated by extruding the hydrated liposomes through a polycarbonate filter (Millipore-Isopore^TM^) with a pore size of 0.1 µm for 20 cycles. The fluorophores ANTS (8-aminonaphthalene-1,3,6-trisulfonic acid, disodium salt) and DPX (p-xylene-bis-pyridinium bromide) were acquired from Molecular Probes (Eugene, OR, USA). The size and polydispersity of the liposomes were determined using a Zetasizer (Zetasizer NANO, Malvern Instruments, Herrenberg, Germany). The ANTS/DPX-loaded lipid vesicles of defined size were separated from the free fluorescent dye via exclusion chromatography using a column packed with a Sephadex^TM^ G-75 (Amersham Biosciences, Buckinghamshire, UK) fine gel swollen in an iso-osmotic buffer (10 mM Hepes, 140 mM NaCl, pH 7.4). The phospholipid concentration was determined through phosphate analysis [53]. The fluorescence emission from the ANTS/DPX loaded lipid vesicles was recorded at 37 °C with an excitation wavelength of 360 nm and an emission wavelength of 530 nm, using monochromators with a slit width of 10 nm. A final lipid concentration of 50 µM was used for these measurements. The fluorescence emission was recorded over time, both before and after the incremental addition of peptides in concentrations ranging from 0.25 to 16 μM, corresponding to the peptide-to-lipid molar ratios from 1:200 to 1:3.125. These measurements were conducted on a VARIAN Cary Eclipse fluorescence spectrophotometer with Cary Eclipse Software version WIN FLR (Agilent, Vienna, Austria). The percentage of leakage was calculated using the formula: IF = (F − F0)/(Fmax − F0), where F represents the measured fluorescence, F0 is the initial fluorescence without peptide, and Fmax is the fluorescence corresponding to the 100% leakage achieved by adding 20 µL of 10% Triton X-100.

For the DSC measurements, the lipid films composed of a mixture of POPE and POPG in a 3:1 molar ratio were prepared with a total lipid weight of 1 mg. These films were hydrated using a Napi buffer (20 mM Na_2_HPO_4_/NaH_2_PO_4_ and 130 mM NaCl, pH = 7.4 (Merck, Vienna, Austria)) following a previously established protocol [53]. The DSC measurements were conducted using a Nano-DSC high-sensitivity differential scanning calorimeter (Waters, Eschborn, Germany), and data analysis was performed using the NanoAnalyze software version 3.8.0, provided by the manufacturer. The liposomes at a concentration of 1 mg/mL were subjected to DSC analysis both with and without peptides, at lipid-to-peptide molar ratios of 25:1 and 100:1. Six scans, involving alternating heating and cooling, were performed for each sample, covering a temperature range from 1 to 75 °C with a heating rate of 0.5 °C per minute. The Napi buffer was used as a reference in these measurements.

### 4.4. Validation of MTB Recovery Efficacy from Clinical Samples

An observational non-interventional evaluation study was conducted according to the standards of the STARD statement for reporting studies of diagnostic accuracy (http://www.stard-statement.org/, accessed on 1 September 2023) on samples obtained from patients presenting at St. George’s Hospital in London, UK, after providing written consent, or at the Pisa University Hospital Microbiology Unit in Italy, and approved by the Ethics committee of the Tuscany North West Vast Area for Clinical Trials. The recruitment and inclusion were based on the provision of informed consent, plus the presenting symptoms indicating a possible or previously diagnosed tuberculosis via MTB culture, GeneXpert PCR (Cepheid, Sunnyvale, CA, USA), or Quantiferon (QuestDiagnostics, London, UK) latency testing. People unable to provide consent, under detention, or unable to produce a sample were excluded. All samples were blinded, and outcomes were analysed retrospectively by the clinical teams. Samples including sputum, bronchial lavage (BAL), pleural fluid, cerebral spinal fluid (CSF), aspirate (pus, lymph tissue), biopsy material, faeces, or urine were not frozen at any time and their storage prior to processing was at 4 °C. All samples were processed within 72 h of receipt. Before decontamination processing, faecal samples (1 g) were emulsified in 5 mL of sterile phosphate-buffered saline and filtered through a 40 µm filter (Falcon, CO, USA), and respiratory samples were solubilised with equal volumes of sputasol (Oxoid, UK) for 10 min at RT. All samples (including urines and aspirates) were then pelleted by centrifuged at 1700× *g* for 15 min and the supernatant was discarded. Samples were then split equally and decontaminated using either a standardised routine reference method of 15 min treatment in 2% NaOH followed by neutralisation in phosphate buffer, or suspended and gently agitated overnight at 37 °C in 10 mL of TiKa-Kic Medium, containing 10 μg/mL T14D, as previously described [7]. After decontamination, samples were centrifuged at 1700× *g* for 15 min, and the pellet was added to MGIT + PANTA tubes for the reference arm culture and MGIT + PANTA + TB08D (1 μg/mL) tubes for the index arm culture. All cultures were incubated and continually monitored for growth for at least 72 days in an MGIT960 (Becton Dickinson, UK). Cultures showing acid-fast staining were either sent to a national reference laboratory for confirmatory identification or confirmed using the MTB-specific RD9 qPCR, as previously described [8], performed on gDNA extracted using the QiaPrep kit (Qiagen, Manchester, UK) to the manufacturer’s instructions, or the MGIT TBc Identification Test (Becton Dickinson, Franklin Lakes, NJ, USA), or the MALDI-TOF (Bruker, Milano, Italy) for some non-MTB identifications.

## Figures and Tables

**Table 1:** Hypergeometric probabilities (Hp) that significant convergence (*p* < 0.05; Functionality factor > 1, marked in yellow, ns = not significant, marked in blue) exists between Genesets 1i/r-8i/r and a range of known differentially regulated MTB transcriptomic profiles. (Further Hp comparisons are available in Supplementary File S1, Table S3).

Transcriptomic Profiles	MTB 0 h vs. 48 h			bTB 0 h vs. 48 h		MTB 48 h vs. 48 h		bTB 48 h vs. 48 h
	0 μg/mL	1 μg/mL	10 μg/mL	0 μg/mL	10 μg/mL	1 μg/mL	10 μg/mL	10 μg/mL
	Repressed after 48 h					Repressed Relative to Control		
	Geneset 1r	Geneset 2r	Geneset 3r	Geneset 4r	Geneset 5r	Geneset 6r	Geneset 7r	Geneset 8r
Cholesterol use and cell wall synthesis (TcrA: Rv0602c)	ns	ns	1.43 × 10^−2^	ns	ns	4.09 × 10 ^−3^	4.34 × 10 ^−6^	9.58 × 10 ^−5^
Infection persistence (MprA: Rv0981)	ns	ns	ns	ns	2.09 × 10^−3^	ns	3.91 × 10 ^−2^	2.09 × 10 ^−2^
Growth and cord formation (PhoP: Rv0757)	ns	ns	ns	ns	ns	2.16 × 10 ^−3^	9.15 × 10 ^−3^	4.75 × 10 ^−2^
Controls growth (TcrS: Rv1032c)	ns	ns	ns	ns	ns	ns	7.95 × 10 ^−3^	1.51 × 10 ^−2^
Controls dormancy (dosR: Rv3133c)	2.16 × 10^−15^	3.81 × 10^−13^	8.48 × 10^−15^	3.95 × 10^−14^	8.48 × 10^−13^	ns	ns	ns
Lipid metabolism and transport (mce3: Rv1963c)	2.96 × 10^−3^	1.88 × 10^−4^	3.55 × 10^−10^	ns	1.26 × 10^−3^	ns	2.13 × 10 ^−15^	4.59 × 10^−2^
Late stationary and dormancy (sigI: Rv1189)	3.52 × 10^−3^	4.62 ×10^−3^	2.46 × 10^−4^	ns	ns	ns	5.38 × 10 ^−3^	ns
Non-culturable MTB profile (Induced geneset)	3.34 × 10^−38^	6.63 × 10^−38^	1.11 × 10^−34^	2.80 × 10 ^−38^	4.21 × 10 ^−34^	ns	3.16 × 10 ^−5^	7.58 × 10 ^−6^
Non-culturable MTB profile (Repessed geneset)	8.07 × 10^−19^	1.34 × 10^−19^	1.13 × 10^−41^	3.96 × 10 ^−14^	5.04 × 10 ^−24^	8.71 × 10 ^−3^	2.37 × 10 ^−36^	3.10 × 10 ^−31^
MTB Persiter profile (Induced geneset)	4.82 × 10^−32^	1.21 × 10^−34^	2.00 × 10^−32^	4.55 × 10 ^−32^	1.16 × 10 ^−33^	ns	1.46 × 10 ^−2^	4.02 × 10 ^−4^
Enduring Hypoxic Response (Induced geneset)	1.86 × 10^−47^	1.21 × 10^−45^	1.80 × 10^−45^	5.54 × 10 ^−47^	1.02 × 10 ^−39^	ns	ns	ns
Cholesterol uptake and metabolism: (KstR: Rv3574)	1.20 × 10^−13^	7.57 × 10^−15^	1.74 × 10^−12^	1.74 × 10 ^−12^	1.46 × 10 ^−13^	ns	ns	ns
Cell division and cell wall synthesis (MtrA: Rv3246c)	7.28 × 10 ^−4^	1.10 × 10 ^−2^	1.82 × 10 ^−4^	2.14 × 10 ^−2^	4.28 × 10 ^−4^	ns	ns	ns
	**Induced after 48 h**					**Induced Relative to Control**		
	**Geneset-1i**	**Geneset-2i**	**Geneset 3i**	**Geneset-4i**	**Geneset-5i**	**Geneset-6i**	**Geneset-7i**	**Geneset-8i**
Non-culturable MTB profile (Induced geneset)	3.08 × 10 ^−13^	2.79 × 10 ^−18^	6.14 × 10 ^−21^	3.80 × 10 ^−15^	7.91 × 10 ^−19^	ns	ns	ns
Non-culturable MTB profile (Repessed geneset)	3.42 × 10 ^−14^	7.60 × 10 ^−33^	2.62 × 10 ^−42^	2.74 × 10 ^−9^	6.84 × 10 ^−37^	ns	ns	ns
Starvation response	2.21 × 10 ^−3^	1.73 × 10 ^−8^	2.55 × 10 ^−24^	4.27 × 10 ^−2^	1.38 × 10 ^−24^	ns	ns	5.10 × 10^−4^
Aerobic Metabolism	2.93 × 10 ^−14^	2.38 × 10 ^−31^	2.10 × 10 ^−42^	2.74 × 10 ^−9^	1.90 × 10 ^−37^	ns	ns	ns
Reactivation from chronic infection (whiB5 regulon)	ns	ns	ns	ns	ns	ns	7.59 × 10 ^−3^	2.88 × 10 ^−2^
Triclosan MTB growth inhibitor	ns	ns	ns	ns	ns	ns	ns	ns
Heat Shock Response	ns	ns	ns	ns	ns	ns	ns	ns

**Table 2:** Total samples positive for MTB or non-tuberculosis mycobacterial (NTM). Smear-ve represents results from routine direct Zeihl Neelsen stain microscopy. FNA = Fine Needle Aspirate, Pl.fl. = pleural fluid, Bx. = Biopsy, BAL = Bronchial Alveolar Lavage. Figures in () represent average TTP in days for each total category: Figures in {} represent average decrease in TTP seen by the improved method in days.

Isolate	Sample	Total Positive by Index Only	Positive by Index and Reference			Total Positive by Reference Only
			Total Index Improved	Total Equal	Total Reference Improved	
MTB	Sputum Smear +ve	2 (13)	17 {3}	3	2 {5}	0
	Sputum Smear −ve	8 (26)	1 {46}	0	2 {3}	0
	Faeces/Urine	14 (29)	1 {33}	0	0	0
	FNA/Bx./Pl.fl.	2 (33)	2 {8}	0	2 {18}	0
NTM	Sputum Smear +ve	0	0	0	0	0
	Sputum Smear −ve	4 (8)	3 {12}	0	0	0
	Faeces/Urine	3 (46)	0	0	0	0
	BAL	1 (12)	0	0	0	0

## Data Availability

Data are contained within the article and supplementary materials.

## Supplementary Materials

The supporting information can be downloaded at: https://www.mdpi.com/article/10.3390/ijms242417555/s1.
