# Supplementary material for: Specific Cationic Antimicrobial Peptides Enhance the Recovery of Low-Load Quiescent Mycobacterium tuberculosis in Routine Diagnostics
Source: Int J Mol Sci. 2023 Dec 16;24(24):17555. doi: 10.3390/ijms242417555 (PMC10743970; doi:10.3390/ijms242417555)
Supplement: Supplementary file 1 [file ijms-24-17555-s001.zip › Supplementary Figures S1-S5.docx]

***Figure S1.***

***Bright field x50 magnification of BCG colonies grown for 6 weeks at 37°C.***

***A. 7GCO+ medium alone B. 7GCO+20μg/ml T14D
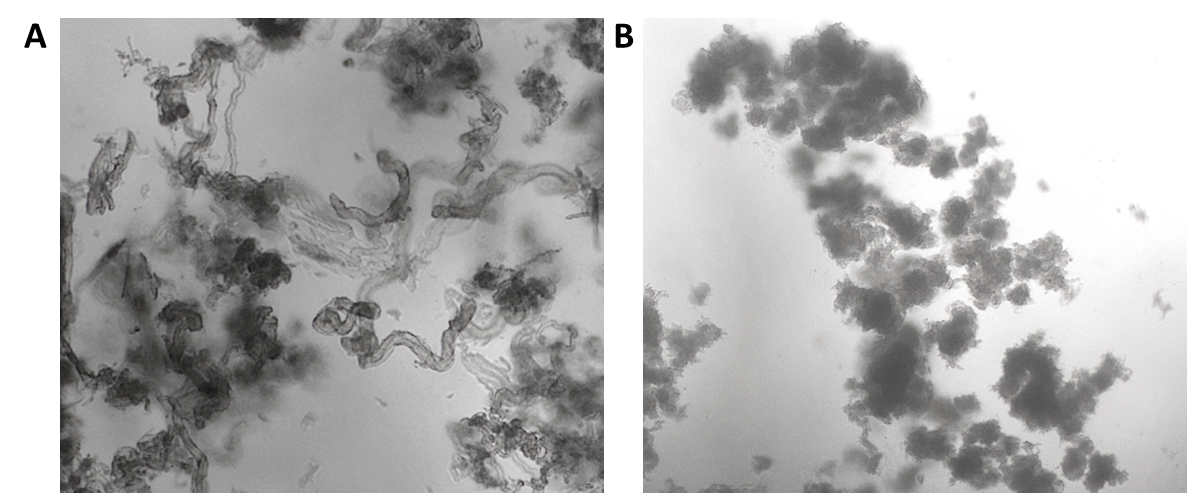
***

***Figure S2. Survival plot of MTB positivity in samples parallel processed with Index and Reference Standard protocols***

**

*
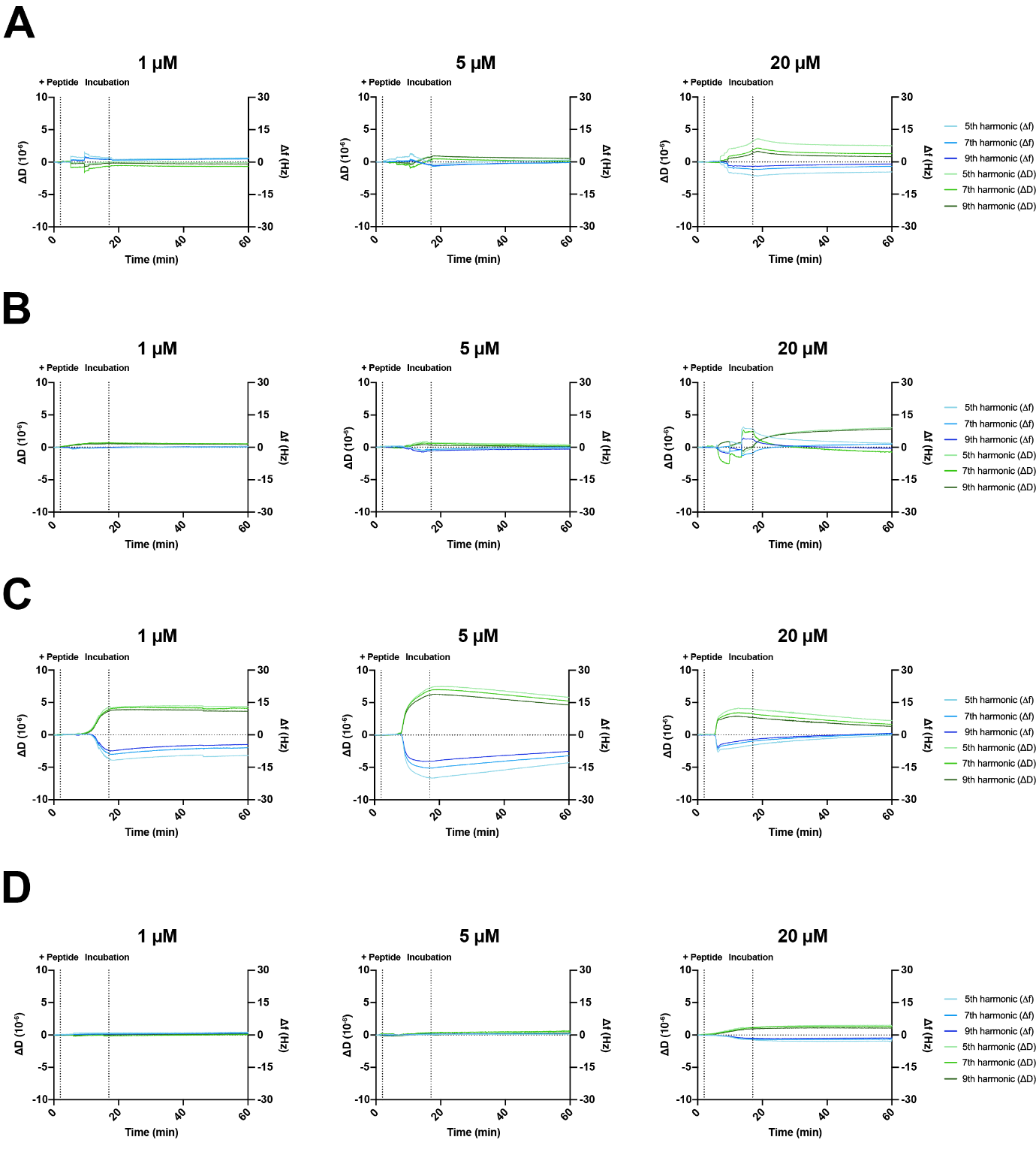
*

*Figure S3 -* *Representative QCM-D time profiles of the 5^th^, 7^th^, and 9^th^ harmonics for peptides* ***(A)*** *T14L,* ***(B)*** *T14D,* ***(C)*** *TB08D, and* ***(D)*** *D02L over POPC bilayers treated with 1 - 20μM peptides.*

*
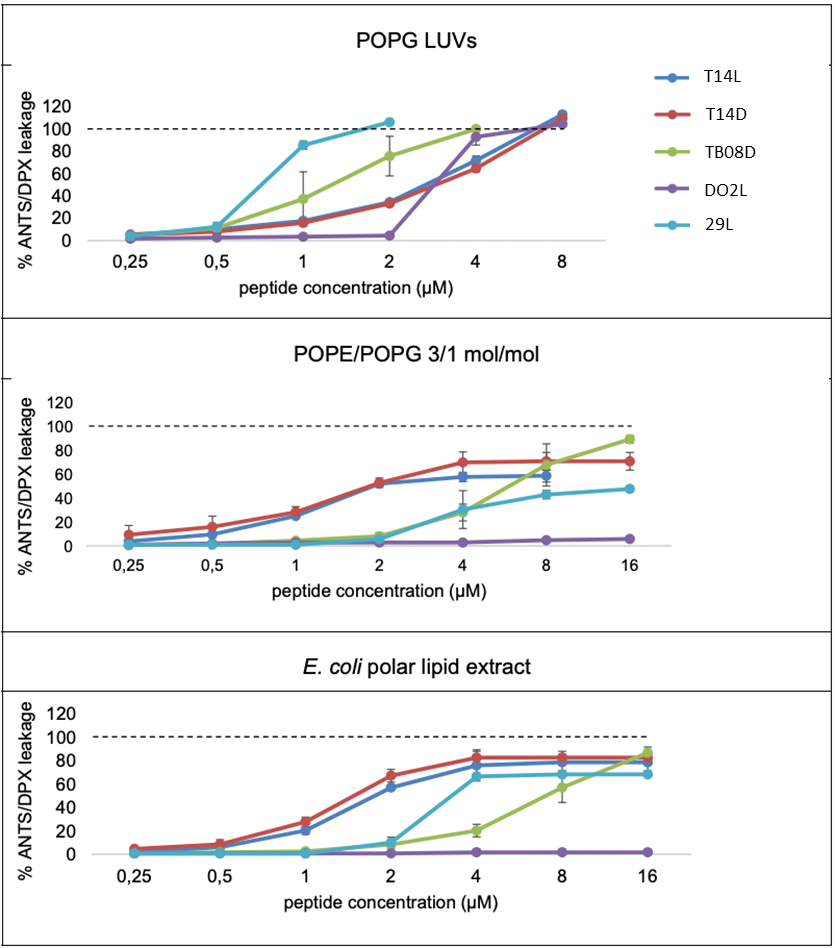
*

*Figure S4. Comparison of* *leakage caused by peptides on POPG,POPE/POPG, E.coli polar extract liposomes.*

*
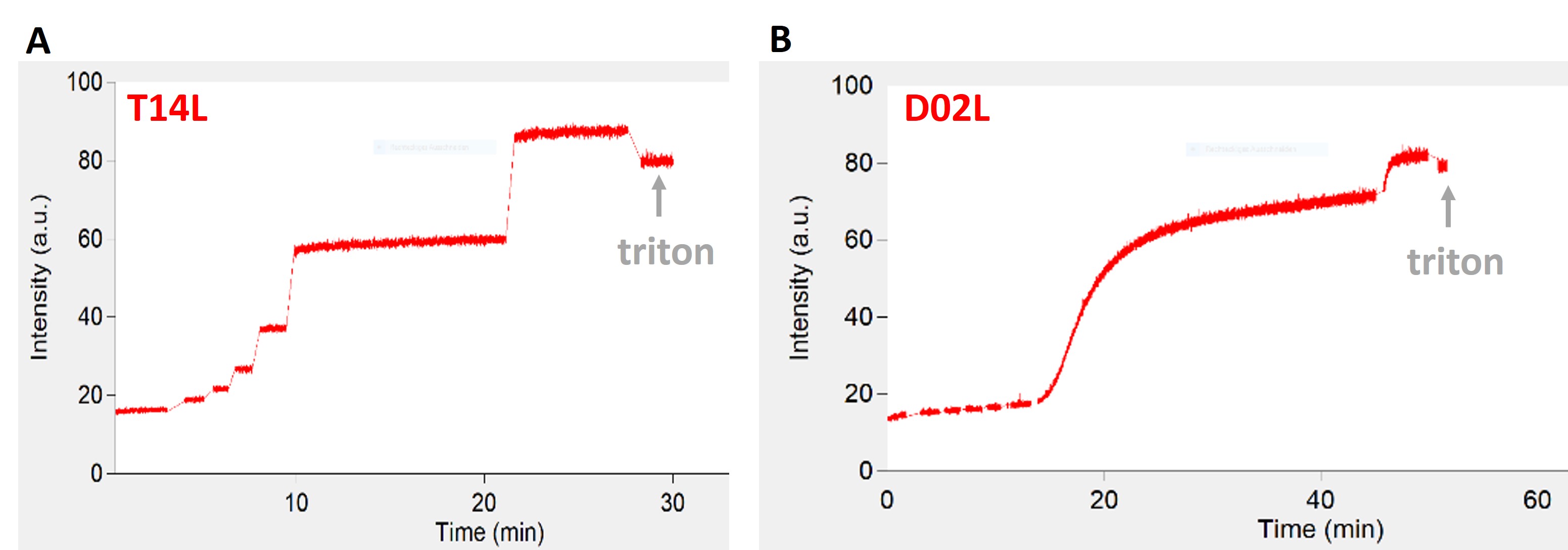
*

*Figure S5.* *Leakage of POPG liposomes by A) T14L and B) D02L*

*The fluorescence of ANTS released from POPG liposomes was recorded while adding increasing amounts of peptides starting from the untreated samples to samples titrated with peptides concentrations ranging from 0.25 to 16 μM, corresponding to a lipid to peptide molar ratio from 200:1 to 3:1. 100% leakage was derived by adding 10 % Triton X-100. Experiments were performed twice.*
